# Performance management in complex adaptive systems: a conceptual framework for health systems

**DOI:** 10.1136/bmjgh-2021-005582

**Published:** 2021-07-29

**Authors:** Tom Newton-Lewis, Wolfgang Munar, Tata Chanturidze

**Affiliations:** 1 Freelance Health Systems Consultant, Oxford, UK; 2 Department of Global Health, George Washington University Milken Institute of Public Health, Washington, District of Columbia, USA; 3 Health Practice, Oxford Policy Management, Oxford, UK

**Keywords:** health systems, health policy

## Abstract

Existing performance management approaches in health systems in low-income and middle-income countries are generally ineffective at driving organisational-level and population-level outcomes. They are largely directive: they try to control behaviour using targets, performance monitoring, incentives and answerability to hierarchies. In contrast, enabling approaches aim to leverage intrinsic motivation, foster collective responsibility, and empower teams to self-organise and use data for shared sensemaking and decision-making.

The current evidence base is too limited to guide reforms to strengthen performance management in a particular context. Further, existing conceptual frameworks are undertheorised and do not consider the complexity of dynamic, multilevel health systems. As a result, they are not able to guide reforms, particularly on the contextually appropriate balance between directive and enabling approaches. This paper presents a framework that attempts to situate performance management within complex adaptive systems. Building on theoretical and empirical literature across disciplines, it identifies interdependencies between organisational performance management, organisational culture and software, system-level performance management, and the system-derived enabling environment. It uses these interdependencies to identify when more directive or enabling approaches may be more appropriate. The framework is intended to help those working to strengthen performance management to achieve greater effectiveness in organisational and system performance. The paper provides insights from the literature and examples of pitfalls and successes to aid this thinking. The complexity of the framework and the interdependencies it describes reinforce that there is no one-size-fits-all blueprint for performance management, and interventions must be carefully calibrated to the health system context.

**Summary box**

- Performance management approaches in many low-income and middle-income country health systems are largely directive, aiming to control behaviour using targets, performance monitoring, incentives, and answerability to hierarchies.
- The complex, dynamic, multilevel nature of health systems makes outcomes difficult to control, so directive approaches to performance management need to be balanced with enabling approaches that foster collective responsibility and empower teams to self-organise and use data for shared sensemaking and decision-making.
- This paper sets out a conceptual framework that identifies the factors that determine the appropriate balance between directive and enabling approaches to performance management in a given context.

## Introduction

To accelerate progress towards Universal Health Coverage and the Sustainable Development Goals at a time of constrained resources, global health actors have increasingly focused on the performance of healthcare providers^1^ and approaches to managing that performance.^2^

A recent evidence gap map on performance management in primary healthcare in low-income and middle-income countries (LMICs) suggests the existing approaches—and interventions to support them—are often unsuccessful at driving organisational and population-level outcomes.^3^ This mirrors findings in the broader public sector management and human resource management (HRM) literature, which also report unintended and sometimes negative effects from performance management systems.^4–6^

Frameworks for considering the performance of health systems conceptualise performance management as a ‘continuous process of establishing targets, monitoring performance against those targets, and implementing and adapting improvement efforts’,^7^ undertaken within facilities (or equivalent, such as primary care teams)—the meso-level, organisational-level of the health system. This aligns with its framing within the public management literature. For example, Pollitt^8^ identifies three components of the cyclical translation of targets into performance outcomes:

- Incentive systems: positive rewards and negative sanctions that incentivise individuals within the organisation to work towards goals.
- Implementation support: tactics used by organisations to achieve performance goals.
- Performance measurement, feedback, and sensemaking: the processes through which performance data are collected, monitored, synthesised, and analysed.

In this conceptualisation, the cyclical nature of performance management relates to how performance results are fed back to healthcare providers and facilities to inform process and service improvements, as well as (in the longer term) to support organisational learning effects such as new strategies and services.

The evidence base is insufficient to guide what incentive systems, implementation support strategies, and sensemaking strategies should look like in a particular context. Evidence is largely limited to studies on the effects of a narrow subset of implementation support strategies (such as in-service training) and financial incentive systems (such as pay-for-performance) on immediate individual outcomes (such as provider knowledge).^3^ There are far fewer studies on other strategies (eg, audit and feedback) or distal outcomes (such as effective coverage and gains in health and/or equity).

Pollitt’s performance cycle framework is not able to guide how to reform its components to strengthen the means, motives and opportunity (MMO) required for individuals^9^ and organisations to perform. In particular, the framework is agnostic regarding the main debate in the public management and organisational behaviour literature: should performance management be more enabling or more directive?^10–14^

Directive approaches treat performance management as a principal-agent problem between workers who are predominantly extrinsically motivated and managers who aim to control their behaviour through targets, vertical accountability, ‘carrot-and-stick’ incentives, and the use of data to monitor compliance through audit style approaches.^15^ Directive approaches characterise how performance management is undertaken in the health sector of many LMICs.^9 16–18^

In contrast, enabling approaches treat workers as stewards,^19^ and assume that workers have intrinsic motivation aligned with health system goals and need to be encouraged and developed rather than measured, incentivised and coerced. Under such conditions, performance emerges if workers have agency and an enabling environment.^20 21^ Enabling approaches emphasise team based incentives, self-organisation, and the shared sensemaking of data via iterative cycles of reflection and learning to foster collective responsibility.^22–26^

Directive vs enabling is a continuum,^11^ and a contextually appropriate balance is required.^14^ Judging this balance is challenging when performance is emergent and results from workers’ agency and motivation, their organisational environments, and their interaction with their external context. Health systems are complex and adaptive: performance outcomes arise from interactions between many interconnected system actors and their ability to adapt to pressures for change. Such conditions make health systems inherently non-linear and unpredictable.^27–29^

Despite literature examples of how performance management interfaces with these and other dimensions of complexity, to the best of our knowledge no existing conceptual framework situates health performance management within complex adaptive systems. The framework presented in this paper aims to characterise the elements within a performance management system and their interdependencies, particularly with the actions of system-level actors who themselves undertake management tasks. Supported by documented examples of success and pitfalls in the literature, we aim to provide a basis for informing the design of performance management reforms and guiding decision makers on achieving a contextually appropriate balance between directive and enabling approaches. This is consistent with the uses of complexity theory in public administration^12^ and health services^26^; it also addresses some concerns.^13^

### The framework

#### Process for development

To develop the framework, we first undertook an integrative literature review. This is an appropriate method to critically review and synthesise the literature on emerging topics to reconceptualise an issue and generate new frameworks.^30 31^ The intention was to combine perspectives on how complexity has been considered in the design, implementation and evaluation of performance management systems from across disciplines (health systems, public management and HRM), rather than systematically covering all articles ever published on the topic. Literature was sourced from existing systematic reviews of the evidence,^3^ a Google Scholar search for ‘complex performance management’ and similar combinations, and a snowball approach from references cited in reviewed papers. Data on how complexity had been conceptualised was abstracted, along with findings from empirical studies. This was synthesised into a reconceptualised framework.

As a starting-point, the framework took the traditional organisational performance management cycle model of Pollitt^8^ and others^32–34^—and its adaptation to multilevel systems^28^—from the public sector management literature. We extended the model based on the theoretical literature from health systems, leveraging recent conceptual advances in how systems performance depends on the dynamic between ‘hardware’ inputs into service delivery (such as supplies, human resources, and infrastructure) and ‘software’ that influences organisational culture and individual behaviour.^35^ This was augmented with a review of the latest literature on performance management from the field of HRM,^6^ including as it applies to health.^18^ Empirical evidence from the health systems literature was reviewed to detail the elements and interactions of the framework, including on how system-level performance management impacts on facilities^36^ and how software within facilities impacts on the performance management cycle.^17 37–39^

Given the intention to motivate the practical understanding of the framework, seven purposively selected, unpublished empirical case studies were developed that covered a range of directive and enabling performance management systems and approaches, to show how they affected different elements and interactions of the framework and provide examples of pitfalls and successes. This includes three examples of internationally recognised performance management systems in high-income countries (UK, Sweden and Italy) and four on performance management interventions in LMICs (results-based financing in Nigeria, performance accountability mechanisms in India, a supranational performance management intervention in El Salvador, and district governance mechanisms in South Africa). The findings from these case studies, along with examples from the broader empirical literature reviewed, are used to illustrate the framework below.

#### Overview

**Figure 1:**
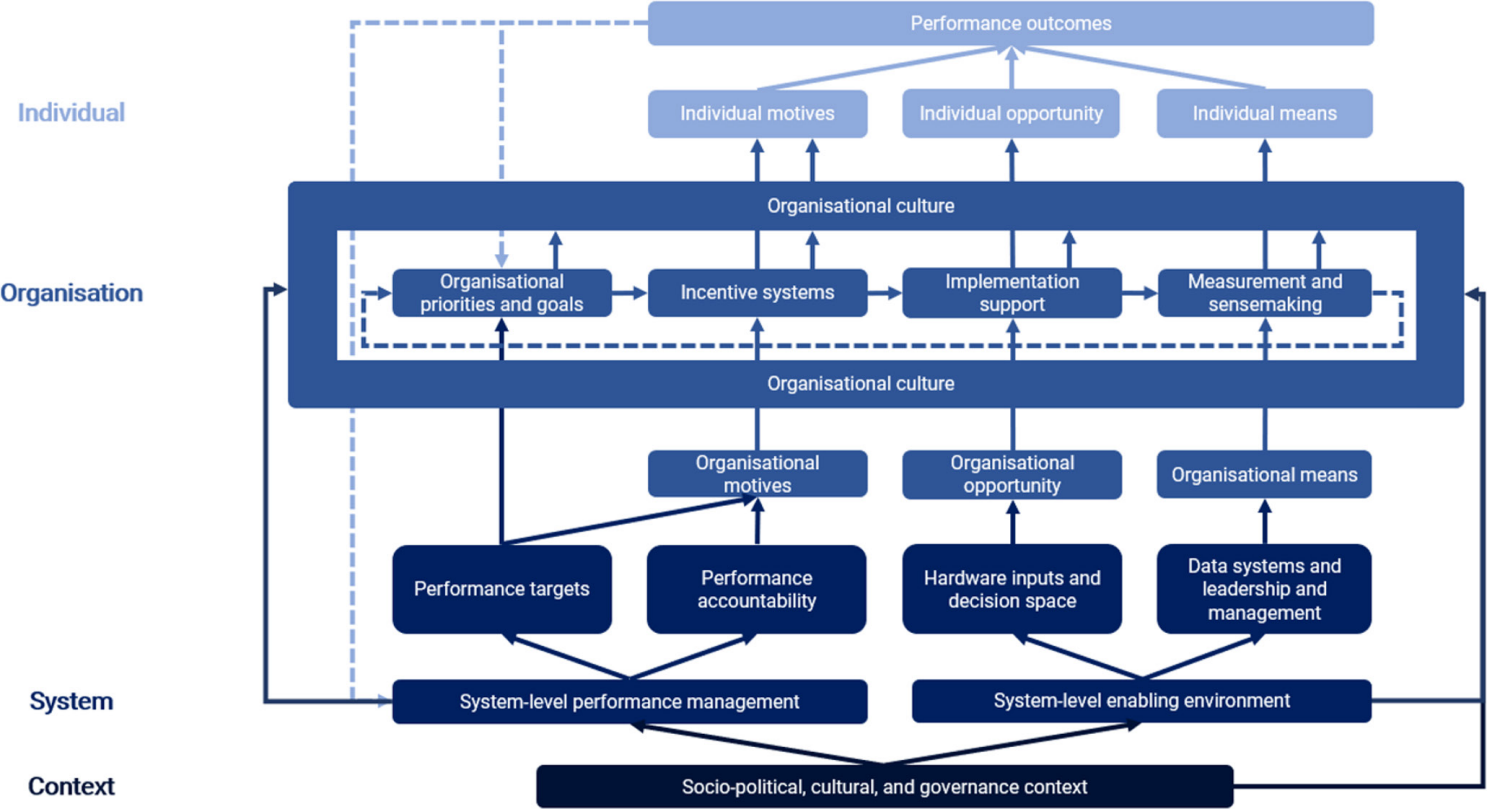
Conceptual framework for performance management in complex adaptive health systems.

The framework is visualised in figure 1. Individual performance occurs if individuals have the required MMO.^9^ This framing has been extended to the organisational level whereby ‘means’ relates to an organisation’s cognitive and behavioural capacity to review and interpret performance data and design and deploy appropriate strategies; ‘motives’ refers to the collective intention to work towards performance goals; and ‘opportunity’ relates to the availability of resources and agency to achieve targets.

System change is hypothesised to be multilevel, with performance emerging from relational interactions between individuals, connections within organisational boundaries, and networks of system elements. This makes system outcomes non-linear and unpredictable.^12 27–29^ In general, in high complexity systems, directive approaches to performance management (which try to mandate outcomes and assume a degree of mechanistic linearity) are likely to be less effective than enabling approaches, which create a conducive environment for high performance to emerge from relational interactions.

Within this, the framework visualises three interactions between the performance management cycle and the broader health system. Their implications for the balance between directive and enabling approaches are discussed in detail below.

- Performance of organisations is ‘managed’ by system-level actors (eg, a Ministry of Health) and enacted through target-setting and accountability relationships. System actions influence the organisational performance management cycle by triggering organisational motives.
- Adequate resources, decision space and data—as part of an enabling environment—provide opportunities and means to undertake performance management.
- The impact of the performance management cycle on individual MMO (and therefore performance) is mediated by organisational culture, itself influenced by system-level actions and the broader sociopolitical, cultural and governance context in which they are embedded.

#### System-level performance management

To achieve system-level goals, system agents undertake performance management of organisational units within them. In most LMICs, this is undertaken through directive, audit-style approaches.^16–18^ It has been argued that these mirror broader governance trends favouring the application and extension of protocols of financial accountability to public institutions, as managers, regulators and politicians attempt ‘at a distance’ control of complex systems.^40–42^

Most systems operationalise this through centrally set targets.^36^ For example, in the UK’s National Health Service, the performance of Clinical Commissioning Groups is assessed against 77 indicators in the Quality and Outcomes Framework.^43^

Potential pitfalls arise from how performance targets are set. First, targets are often unrealistic given the resources and decision space available to teams, creating incentives to game the system and manipulate data.^5 44 45^ Second, people-centred healthcare cannot be easily reduced to quantitative benchmarks^18 42^ and often targets can focus on outputs at the expense of improvement and outcome indicators, incentivising a focus away from quality of care.^45^ The literature acknowledges the need for an appropriate balance between process, output, and outcome targets,^46^ for example through a balanced scorecard approach.^16^ Thirdly, centralised targets can have limited legitimacy among providers; whereas participatory target-setting can increase trust, teamwork and cooperation.^6^

Directive approaches have also been criticised when top–down targets do not respond to local priorities or fail to create opportunities for the emergence of collaborative work among health system actors.^18^ Where there is uncertainty regarding how desired outcomes should be achieved, minimum specification approaches—a few simple, flexible rules, combined with direction pointing—may be more effective than prescriptive targets, as they allow for local innovation and contextually appropriate self-organisation.^47^ Target flexibility in response to local contexts can help. For example, in Sweden, the 21 districts tailor the national performance management system at the regional level.^48^

Performance targets are expected to form the basis of an accountability relationship, giving organisations the motive to perform. Hierarchical and transactional accountability relationships may exacerbate the risk of unintended consequences arising from performance targets. For example, in India, punitive treatment of staff in facilities that did not meet system-mandated targets, such as salaries being withheld, led to coordinated and systematic falsification of data.^49^ The literature shows how ‘accountability overloads’ can ‘create bureaucratic compliance, demotivation, reduced efficiency and effectiveness and limited space for innovation’.^38^ This contrasts with constructive accountability, which promotes collective responsibility and a culture of learning rather than blame.^39^ Strict performance accountability mechanisms inherent in directive performance management systems may be more appropriate when there is limited existing accountability in the system.

Performance-based financing (PBF) is commonly used to operationalise accountability. For example, in El Salvador, under the Salud Mesoamerica Initiative (SMI), when service provider teams achieve 80% or more of their targets, they receive team-based, in-kind rewards worth up to US$1000.^50^ The literature shows the challenges of designing an approach that effectively promotes performance without unintended consequences.^51^

#### System-level enabling environment

Performance management requires organisations to have the opportunity to perform, with sufficient agency over outcomes.^52^ This requires adequate hardware resources (eg, infrastructure, supplies and human resources). Otherwise, improvements may be limited to efficiency gains and performance management approaches are more likely to be unrealistic, incentivising gaming.

In addition, the health systems literature identifies that organisations often have insufficient decision space, lacking autonomy in health planning, budget allocation and HRM.^53 54^ The experience of Nigeria shows that additional resources and control arising from PBF schemes, rather than incentivisation, can be a primary driver of impact.^55^ In El Salvador, the highest-performing primary healthcare teams use their autonomy to self-organise to provide outreach services to the hardest-to-reach communities.^50 56^

Measurement and data are key for organisations to undertake performance management. Evidence from Mozambique, Rwanda and Zambia shows that data-driven quality improvement using ‘plan–do–study–act’ cycles can improve service delivery.^57^ Effectiveness will be shaped by whether data review processes are used in directive (for audit-style monitoring and control) or enabling (for collective sensemaking) ways.

For higher-order learning and whole-system improvement to occur, practical and tacit knowledge needs to flow among system actors and organisations, thus leveraging the power of networks and social connections (eg, learning exchanges and communities of practice). In El Salvador, the highest-performing teams share experience and know-how with the entire community of team leaders, thus turning routine supervision meetings into strategic opportunities for learning and collective sensemaking.^56^

The literature also emphasises the importance of appropriate leadership and management capabilities. The capacity of managers—particularly at the sub-national level—to diagnose problems, identify and implement solutions, and manage performance is increasingly emphasised as crucial to better health system performance.^58 59^ Poor leadership has undermined priority-setting and resource-allocation practices in hospitals in Kenya.^60^ Enabling performance management styles require leaders to have soft skills (eg, communication, trust-building and networking) on top of the hard skills (eg, planning and monitoring) required for directive approaches.^17 23^

#### Organisational culture

The health systems literature emphasises the importance of organisational culture,^61^ including intangible software dimensions^35^ such as power dynamics and shared norms and values, on team and individual behaviour.

Conducive culture—manifested through high levels of teamwork, recognition, and trust, and individuals feeling they receive organisational support and reciprocity^62^ —is crucial for enabling approaches to performance management that require collective organisation. The complex web of relationships within facilities, underpinned by formal and informal power dynamics, can also subvert directive performance management approaches, for example through quid-pro-quo behaviour and political connections undermining management controls.^17 37–39^ This is mirrored in the HRM literature, which shows that the effectiveness of performance management approaches depends on the social context and how users react,^63^ which are in turn influenced by perceptions of fairness, supervisor–supervisee relations, leadership and organisational culture.^6^ Organisational culture has been shown to be amenable to intervention through coaching and mentoring to foster transformational leadership styles that build trust, motivation and teamwork.^64 65^

Performance management approaches can in turn influence organisational culture. For example, micro-practices of social sensemaking within enabling approaches have been shown to improve motivation and collective commitment.^23^ District-level Monitoring and Response Units in South Africa have positively influenced intangible software through facilitating new spaces for more participatory sensemaking.^66^ Conversely, audit style performance management can damage organisational culture, create anxiety, insecurity and mistrust, and undermine commitment, loyalty and performance.^18 42 44^ For example, in India, unrealistic targets led to a defeatist attitude among nurses.^49^

## Conclusions

In the context of the limited effectiveness of existing performance management approaches in LMIC health systems, and the sparse evidence base and lack of a system-based framework to guide reforms, this paper has presented a framework attempting to situate performance management within complex adaptive systems. Building on theoretical and empirical literature across disciplines, this framework has identified interdependencies between organisational performance management cycles, organisational culture, system-level performance management, and the system-derived enabling environment.

In particular, the framework has been used to consider the strengths and weaknesses of directive and enabling approaches in different contexts. Directive approaches (seeking to control behaviour based on targets and accountability relationships) may be more effective where workers are primarily extrinsically motivated, in less complex systems where there is higher certainty over how outcomes should be achieved, where there are sufficient resources and decision space, and where informal relationships do not subvert formal management levers. Enabling approaches (promoting self-organisation and collective sensemaking) may be more effective in contexts of higher complexity and uncertainty and where there are higher levels of trust, teamwork, and intrinsic motivation, as well as appropriate leadership.

Directive and enabling approaches are not ‘either-or’: designers of performance management systems must strive for an appropriate balance between them. The literature indicates a degree of complementarity: directive approaches can fuel short cycles of innovation and improvement, but enabling approaches are necessary for long-term strategic renewal and change.^11 14^ For example, in El Salvador, the successful SMI relied on directive elements (including targets, in-kind incentives, and measurement and audit). It also explicitly promoted social interactions, fostered multidirectional feedback and learning loops that built trust, and delegated the decision space on achieving targets to semi-autonomous teams.^56^

The SMI is also a good example of a comprehensive intervention targeting system-enabling factors, with strong data systems and the use of organisational financial incentives as untied funds to alleviate resource constraints. This is an important reminder of the need to facilitate the emergence of an enabling environment for performance management alongside optimising performance management systems.

The framework’s complexity and its interdependencies reinforce that there is no ‘one-size-fits-all’ blueprint for performance management.^51^ Interventions must be carefully calibrated to the context of the health system, the culture of its organisations, and the motivations of its individuals. Failing to engage with context can contribute to well-meaning interventions not having their anticipated effects.^67^ The greater the dissonance between designing a performance management system and the real context in which it is implemented, the more likely it is to trigger perverse, unintended consequences.^5^

This complexity makes strengthening performance management in health systems extremely challenging. Through categorising the interdependencies between system elements, the framework is intended to support those designing performance management reforms to systematically consider the range of factors that are critical in determining optimal approaches and identify complementary interventions that may be required. They should consider the existing balance between directive and enabling approaches against the degree of uncertainty over how targets should be achieved, the current levels of accountability in the system, the sources of motivation of workers, the decision space and hardware resources available, and the organisational culture, data systems and leadership skills that exist. By considering each factor and their interdependencies, actors can minimise perverse unintended consequences while attaining a contextually appropriate balance between directive or enabling approaches.

## Data Availability

There are no data in this work.
